# Corn Steep Liquor as an Efficient Bioresource for Functional Components Production by Biotransformation Technology

**DOI:** 10.3390/foods14132158

**Published:** 2025-06-20

**Authors:** Ying Chang, Xin-Qi Zhao, Xin Zhang, Yan Jiao

**Affiliations:** College of Food and Bioengineering, Qiqihar University, Qiqihar 161006, China; 02512@qqhru.edu.cn (Y.C.);

**Keywords:** plant nutrients, corn steep liquor, biotransformation, low cost, functional components

## Abstract

Corn is an important crop that can be used to produce many bioactive compounds. These functional components have been widely applied in the pharmaceutical, cosmetic, and food industries. Corn steep liquor (CSL) is a by-product of deep processing of corn that contains a lot of protein, peptides, amino acids, vitamins, and other nutrients, which is considered to be a rich and cheap source of plant nutrients. However, CSL is not widely used and factories are required to treat CSL as waste water directly; therefore, the question of how to turn CSL waste into a valuable product is likely to become a hot topic. In order to fully explore the potential utilization value of CSL, this review comprehensively summarizes the structural composition and nutritional characteristics of CSL, and its application and prospect in the biotransformation of industrialized organic acids, polysaccharides, lipids, enzymes, natural pigments, and novel functional components through the microbial fermentation pathway. Furthermore, specific methods for bioconverting various active substances using CSL were proposed, and the influences of various production conditions on the yield of the bioactive substances were fully analyzed and discussed. This article provides a reference for the efficient utilization of corn steep liquor as a by-product of corn processing.

## 1. Introduction

Corn is one of the most important food and feed crops worldwide and a key source of starch and protein resources. The world produces over 1 billion tons of corn annually, accounting for around 35% of global grain output [1]. The primary by-product of corn starch processing, referred to as corn steep liquor (CSL), is produced via a multi-step extraction and concentration process. The soluble solids in CSL constitute approximately 40–50% (*w*/*w*) of its dry weight, making it a valuable source of nutrients and fermentation substrate [2]. CSL is a dark brown liquid with a strong odor, an acidic pH, and a high viscosity which is not suitable for secondary processing and utilization in the food and feed industries [3]. Organic acids, protein, amino acids, vitamins, and sugars are the primary components of CSL, derived from the wet milling procedure of corn [4]. The main chemical constituents of CSL are listed in Table 1. Corn steep liquor is often used as a cheap source of carbon, nitrogen, vitamins, or micronutrients [5,6]. However, because of its high biological oxygen demand (BOD value, 5000~20,000 mg/L) content, CSL is often treated as waste and promptly discarded, resulting in resource waste and environmental pollution [7]. Addressing challenges in existing technologies and developing eco-friendly strategies to convert CSL into a high-value resource remain key research priorities.

As a by-product of the deep processing of corn, CSL has a high nutritional value and may be employed in the fermentation and feed sectors. The protein in CSL makes up 48 percent, along with lactic acid, amino acids, total carbohydrates, and other minerals, including potassium, calcium, and phosphorus [8]. CSL has already been used as an added component of microbial fermentation, feed, antibiotics, fertilizer, growing medium, and soil regulator [9,10,11]. Consequently, corn steep liquor represents a promising biological resource for the development of high-value products, supporting sustainable and eco-friendly industrial practices [12]. Types and yields of protein by-products in wet starch processing of corn are listed in Table 2.

**Figure 1:**
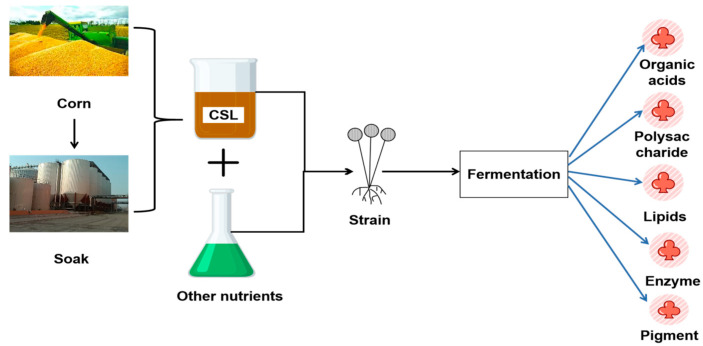
Functional components produced by CSL biotransformation.

Corn steep liquor has a wide variety of application in fermentation engineering, feed engineering, and medicines and includes a significant number of useful compounds [4]. Although CSL currently has been used for producing fermented polysaccharides, pigments, enzymes, and many other products, the current supply of CSL significantly surpasses market demand, primarily driven by the recent accelerated expansion of corn processing facilities. Therefore, the latest development trend of corn pulp is to explore new resource utilization methods and fully realize the industrialization of efficient utilization of CSL. In order to provide an advanced and systematic strategy to effectively develop organic acids, polysaccharides, lipids, enzymes, and natural colors through the bioconversion of corn steep liquor, this article makes a detailed summary of the research in this aspect as well as some new methods for the comprehensive and efficient utilization of CSL that have been proposed. Figure 1 provides a brief overview of the functional components produced by CSL biotransformation.

## 2. Organic Acids Production by CSL Bioconversion

Organic acids, primarily derived from natural metabolic processes, have found extensive applications in the food, pharmaceutical, and bio-based materials industries due to their ability to biodegrade and functional versatility [13]. Organic acids play important roles in the human body. Some natural organic acids, such as citric acid, malic acid, and tartaric acid, have antibacterial, anti-inflammatory, hypoglycemic, antioxidant, and immunomodulatory functions. Organic acids are synthesized by traditional industrial chemistry using a variety of simple chemical units (mainly from fossil sources) [14]. However, the environmental pollution caused by the production process of traditional industrial chemistry has prompted the search for new sustainable resources for the synthesis of organic acids. Due to the negative impacts brought by the chemical production of organic acids, researchers are looking for a new available method, such as microbial production, which is environmentally friendly and sustainable [15,16].

### 2.1. Malic Acid Production by CSL

Malic acid is a C4 dicarboxylic acid widely used as flavor enhancer and acidulant in food and beverage industries [17]. Malic acid is one of the most common organic acids in fruits and is the main acid in many fruits, such as apples, bananas, and lychees [18,19,20]. Also, malic acid is a cyclic intermediate of tricarboxylic acid, an important component of natural fruit juices, and an essential organic acid, making it a preferred low-calorie food additive [21]. Currently, malic acid is mainly produced through chemical transformation of petroleum-derived fumaric or maleic acid [17]. However, recent rising petrochemical prices and concerns of depleting oil resources have generated high interest in producing malic acid from renewable biomass via fermentation. Similarly to other organic acid fermentations, the bioproduction of malic acid is severely constrained by product inhibition, which adversely affects its titer, yield, and productivity, thereby rendering the process economically infeasible. CSL contains the nutrient sources required for malic acid production and can effectively increase the yield during the malic acid fermentation process. For example, polymalic acid (PMA) was produced by ZX-10 soybean shell hydrolysate supplemented with corn soak (CSL). When 300 g/L soybean molasses was used as carbon source, the PMA yield (0.69 g/g) of soybean shell hydrolysate supplemented with 5 to 15 g/L CSL was significantly higher than that of the control supplemented with NH_4_NO_3_ (2 g/L), and a higher productivity of 0.64 g/L·h was obtained in repeated batch fermentation with cell recycle and CSL supplementation. This result indicates that the addition of CSL facilitates the production of PMA from soybean hull hydrolysate with a low source of nitrogen [22].

### 2.2. Citric Acid Production by CSL

Citric acid (2-hydroxypropane-1,2,3-tricarboxylic acid) gets its name from the Latin word citrus, referring to a genus of flowering plants whose fruits include oranges, lemons, limes, and grapefruits. Pure citric acid is a colorless crystalline solid that is highly soluble in water [23]. Citric acid (CA) is a key intermediate in the tricarboxylic acid (TCA) cycle, and the compound is abundant in fruits such as lemons. To date, global demand for CA has been met mainly through fermentation processes. Common applications for citric acid include those in the food and beverage, cleaning, medicinal, cosmetic, and other industries [24]. Approximately 70% of the total annual production of 1.5 million tons is used in the food and beverage industry as acidifiers or antioxidants to maintain or enhance the flavor and aroma of fruit juices, ice creams, and jams [25]. Different strains of filamentous fungi, mainly Aspergillus niger strains, are the main species used for mass fermentation of CA from molasses, sucrose, or glucose. However, the use of fungi to produce CA has been associated with the accumulation of large amounts of solid and liquid waste. Therefore, this method of production increases environmental pollution and costs. Therefore, it is essential to optimize citric acid production by finding alternatives which are more economical, environmentally friendly, and yield higher than current methods [26]. Yersinia lipolytica SWJ-1b was cultured with medium containing corn extract (CSL) instead of yeast extract [27]. The citric acid (CA) produced by the strain in the medium containing 1.0 g/L CSL was 27.5 g/L, which was 1.24 times higher than that in the control medium containing yeast extract. The results indicate that CSL can be used as an alternative source of organic nitrogen. In addition, CSL significantly stimulates carbon source utilization and nutrient absorption, leading to enhanced citric acid production and higher final titers. These results demonstrate the potential application of CSL in low-cost CA production on a commercial scale.

### 2.3. Lactic Acid Production by CSL

**Figure 2:**
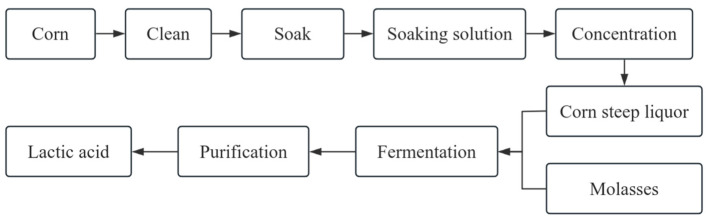
Lactic acid production process by CSL from corn processing.

Lactic acid (LA) is the end product of carbohydrate metabolism [28]. LA has hydroxyl and carboxyl groups and is the most widely distributed hydroxy carboxylic acid in nature, which can participate in a variety of reactions such as oxidation, reduction, condensation, and hydroxyl substitution to prepare various lactic acid derivatives [29]. LA also has a mild acidity and can be added to food to improve its flavor in the food industry [30]. In the fermentation industry, LA is used to control pH and improve the purity of fermentation products [31]. Lactic acid can be produced by chemical synthesis or microbial fermentation. The significant advantage of biofermentation over chemical synthesis is the possibility of using inexpensive raw materials such as whey, molasses, starch waste, sugar beet, cane sugar, and other carbohydrate-rich materials [32,33,34]. However, the efficiency and economy of the final lactic acid fermentation remains an issue in many aspects and the medium composition plays a crucial role in the improvement of the process. Research efforts have focused on finding new sources of effective nutrients to achieve high substrate conversion rates and high yields [35]. Previous studies have reported that Lactobacillus WYZ-L generated 75 g/L of L(+)-lactic acid in fermentations using sugarcane molasses and corn steep liquor without the inclusion of any extra nutrients, with an 85% yield, a maximal productivity of 1.18 g/L per hour, and more than 99% optical clarity [36]. These outcomes revealed that WYZ-L can effectively utilize molasses and corn steep liquid as nutritional sources to produce L(+)-lactic acid through fermentation. Figure 2 displays the process flow chart of lactic acid production by corn steep liquor fermentation.

In order to substitute yeast extract for cost-effective manufacturing, and explore low-cost nitrogen sources, corn steep liquor and ammonium sulfate were mixed with other ingredients for a lactic acid fermentation experiment. The contents of lactose, corn steep liquor, and ammonium sulfate were 55 g/L, 15 g/L, and 5.625 g/L, respectively. The highest level of lactic acid was 18.68 g/L [37]. In order to enhance the fermentation process after the ideal nutritional circumstances for lactic acid production, a second center complex design was carried out to ascertain the optimal production conditions of lactic acid. The highest lactic acid yield, as found by the second-order polynomial regression model, was 5.9 g/L at the optimum temperature and pH of 52.37 °C and 3.96, respectively. It is concluded that addition corn steer liquor to ammonium sulfate can increase the yield of lactic acid. The different types of organic acids produced by corn steep liquor fermentation are summarized in Table 3.

## 3. Polysaccharide Production by CSL Bioconversion

Polysaccharides are widely present in nature and are biological macromolecules with many biological activities. The biological activities and functions of polysaccharides are mostly related to the immune system; they play important roles in living organisms, with antioxidant [38], immune regulation [39], anticoagulant [40], anti-tumor [41], anti-inflammatory [42], antibacterial [43], antiviral [44], etc., properties. The research on polysaccharides has made some advancements in recent years and at least 30 kinds of polysaccharides have been studied clinically for various diseases. But there are still some shortcomings such as their low extraction rate, high cost, and not being to be absorbed by the human body. CSL contains various organic compounds, including polysaccharides as a carbon source, organic acids and vitamins, as well as amino acids and proteins as a nitrogen source, which can be used for microbial fermentation to produce polysaccharides.

*Rhizobium viscosum* polysaccharides were produced by a medium containing sugarcane molasses (60 g/L) and corn steep liquor (10 mL/L) as sole ingredients, this medium allowed the production of 6.1 ± 0.2 g/L polysaccharides, twice the amount produced in the standard medium [45]. Cordyceps militaris polysaccharides were produced through submerged fermentation using a sole corn steep liquor (CSL) medium, the fermentation yield of polysaccharides was 1.185 g/L, which was 20.43% higher than that of the conventional group [46]. The important exopolysaccharide, pullulan, was synthesized from corn steep liquor, and the yield of pullulan was increased by 18% by single-point optimization, and the use of CSL as a nutrient could reduce the production cost of pullulan by one-third [47]. Furthermore, most studies have examined the use of compounds in corn slurry in microbial growth or synthetic media further [48,49,50,51] and few studies have addressed the application of CSL as a functional food or therapeutic agent for health promotion. Currently, most CSL is disposed of due to its enormous production; therefore, it is important to investigate the application of polysaccharides produced from CSL as raw material in food production.

## 4. Lipids Production by CSL Bioconversion

Lipid is one of the important nutrients needed by the human body. Along with protein and carbohydrates, it is known as one of the three major nutrients for productivity and plays an important role in providing energy for the body. The esters and their derivatives produced by the interaction of fatty acids and alcohols are collectively called lipids. Lipids have a wide range of applications, such as in the food, livestock feed, and pharmaceutical industries. Lipids have attracted more attention in recent years [52]. However, the complex extraction process and high extraction cost of lipid compounds limit the research and application. CSL is evaluated as an ideal agricultural feedstock for efficient lipid production [53]. Corn steep liquor contains organic nitrogen, which has been used as a substitute for nitrogen sources in microbial fermentation. In some studies, corn steep liquor was used as the nitrogen source for extracting ester compound culture medium, which not only replaced the expensive yeast extract and peptone and other nutrients, but also significantly promoted cell growth, reduced production costs, and improved production efficiency. It is one of the raw materials for effectively improving the extraction process of lipid compounds [54].

### 4.1. Polyhydroxybutyrate Production by CSL

Polyhydroxybutyrate (PHB) is a kind of bioplastic synthesized by microorganisms, with biodegradability and biocompatibility, which can be widely used in biomedical materials, environmental protection, and other fields. However, one of the problems faced by developing polyhydroxyalkanoates as substitutes for traditional plastics is that their production price is higher than that of petroleum-based synthetic plastics. Therefore, in addition to changing fermentation strategies to control polymer costs, the use of cheaper sources of carbon and nitrogen to reduce production costs is also key to PHB production research.

Corn steep liquor is a by-product of the starch industry and is a cheap alternative to expensive sources of nitrogen. The efficiency of PHB synthesis using corn steep liquor as a cheap nitrogen source was 73% higher than that in normal medium [55]. The maximum yield of PHB was obtained when the mount of molasses was 65.4 g/L and corn steep liquor was 13.2 g/L, and the lower cost of using corn steep liquor compared with other nitrogen source culture media could greatly reduce the production cost and improve the yield [56]. In the above studies, using corn steep liquor to produce fat efficiently can not only improve the utilization rate of industrial waste, but also reduce the production cost; therefore, CSL has prospects for wide applications.

### 4.2. PHA Production by CSL

Polyhydroxy fatty acid ester (PHA) is a general term for a class of polymer polyesters completely synthesized by microorganisms. Because of its biodegradability and biocompatibility, PHA is considered to be an environment-friendly material, which helps to solve the increasingly serious environmental pollution problem. At present, the biggest obstacle to the industrialization of PHA is the high production cost. For this reason, biologists have been working to gradually reduce the production cost of PHA from multiple perspectives, including the cost of substrate, the conversion rate of substrate to product, and the energy consumption of the fermentation production process.

Researchers improved the medium for PHA production and found that the production of PHA using corn steep liquor as a nitrogen source increased the cell growth density by 28%, thus increasing the production of PHA by 39% [57]. A new fermentation medium was used to produce PHA, using gluconate as carbon source and corn steep liquor as a nitrogen source, which reduced the raw material cost by 60%, and established a stable, continuous, efficient, and low-cost PHA production open process [58].

### 4.3. DHA Production by CSL

Docosahexaenoic acid (DHA) is one of the essential fatty acids in human body, which has important functions in both physiological regulation healthcare. In addition, it has special prevention and treatment effects on cardiovascular diseases. However, the material cost required for the production of DHA is high and the process flow is complex, resulting in the low yield and high cost of DHA.

**Figure 3:**
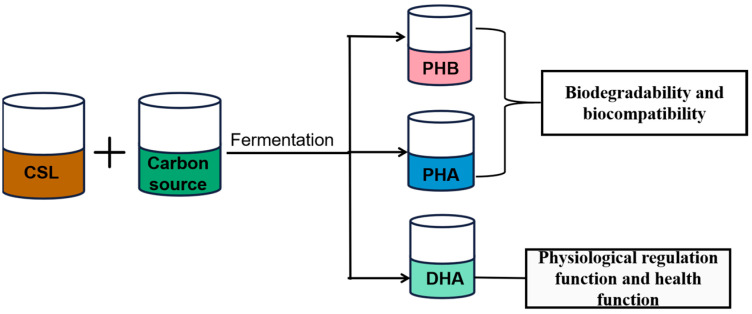
Production process of different kinds of lipids and their functions by corn steep liquor biotransformation.

Due to the production and application status of DHA, many researchers at home and abroad have improved the production process conditions of DHA to optimize the production of DHA. When the corn steep liquor concentration reached 5 g/L and the carbon–nitrogen (C/N) ratio reached 1:2, the oil content, DHA yield, and content reached the highest level, and the cost of using corn steep liquor was significantly lower than that of other types of nitrogen sources [53]. Using starch as the carbon source and corn steep liquor as the nitrogen source for batch culture enabled a maximum biomass concentration, lipid content, and lipid productivity of 5.34 g/L, 24.6%, and 0.016 g/L^−1^·h^−1^, respectively, to be obtained and reduced the fermentation production cost and saved 84% of the process cost [59]. Based on the research results of domestic and foreign scholars, the feasibility of corn steep liquor as a nitrogen source in the production of ester compounds was proved. It is of great significance to promote the synthesis and application of ester compounds and the corn deep processing industry. The production process of different kinds of lipids and their functions by corn steep liquor biotransformation is shown in Figure 3.

## 5. Enzyme Production by CSL Bioconversion

**Figure 4:**
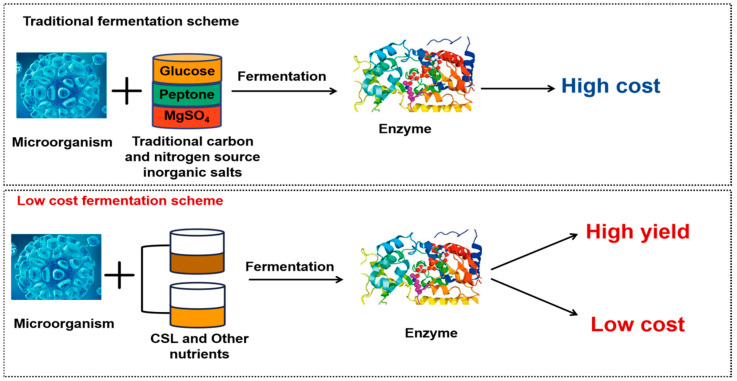
Difference in enzyme production between conventional fermentation and corn steer liquor fermentation.

Enzymes, as highly effective biocatellators, are widely used in industrial applications, such as the pulp and board industry, textiles, biobleaching, food industry, and biofuel industry [60]. Enzymes are obtained from a variety of plant, animal, and microbial sources. Of these, microorganisms are the most efficient source of enzyme production, as they can be easily cultured and have a short life cycle, and enzymes are readily available in a short period of time [61]. Currently, the primary methods for producing enzymes are microbial fermentation and cell culture. Because of the high cost of cultures, cells, and the culture process, it is currently not possible to successfully boost industrial enzyme production, which drives up the cost of enzyme products. However, changing the composition of the culture medium by substituting the original carbon and nitrogen sources for fermentation cultures with comparatively low carbon and nitrogen sources can significantly increase the output of enzymes while also lowering the cost of fermentation. Corn steep liquor, a by-product of the wet processing of maize starch, is a great alternative nitrogen source for strain fermentation because it contains abundant protein, amino acids, and other ingredients that are good for microbial development and enzyme production. For instance, compared with non-induced culture, the addition of 2% CSL significantly increased the laccase production (314.62 + 5.93 IU/mL) and raised the Lac1 gene transcriptional level by 5.6 times [62]. CSL increased L-methioninase production (161.95 U/mL) two-fold compared with that obtained by the Czapek–Dox’s medium (73.92 U/mL) [63]. Figure 4 shows the difference in enzyme production between conventional and corn steer liquor fermentation.

### 5.1. Corn Steep Liquor Fermentation Produces Cellulase

Cellulase is responsible for cellulose degradation by hydrolysing the β-1,4-glycosidic bond [64]. It is a widely used industrial enzyme that has been in commerce for over 30 years, but the enzyme is still a topic of interest in academic research and industry [65]. Cellulose is converted into a simple sugar, glucose, which can be fermented into cellulosic biofuel [66]. Cellulase is widely used in textile, animal food, pharmaceutical, detergent, and paper processing industries [67]. Although cellulase has a wide range of applications, production costs have hampered its development.

Cellulase was produced by fermentation with Streptococcus aureus using waste beer (BSG) and wheat bran (WB) as carbon sources and CSL as a nitrogen source. The results showed that in the valley of beer containing 0.5% fat, 1.2% corn steep liquor medium, a fermentation of 4 d, cellulase production of up to 720 U/L, and using corn steep liquor as the nitrogen source alternative reduced the cost of fermentation [68]. Using bagasse and wheat bran carbon sources and corn steep liquor as nitrogen sources, the cellulase yield reached 2.0 U/mL at day 5 when the addition of wheat bran was 2.0% and the addition of CSL was 0.19% [69]. Using *Bacillus thermophilus SMIA-2* to produce cellulase, they found that in the medium containing bagasse and corn steep liquor, the crystalline cellulase and carboxymethyl cellulase produced by *Bacillus thermophilus* during fermentation of 120 h and 168 h was able to reach 0.83 U/mL and 0.29 U/mL, and the fermentation cost was lower than that of traditional media [70]. Using bagasse as the carbon source and corn steep liquor as the nitrogen source to produce endoglucanase by fermentation of Streptomyces Michais PESB-25, it was found that when the added amount of bagasse was 1.0% and the amount of corn steep liquor was 1.2%, the enzyme yield was highest when the strain fermented for three days, reaching 1.01 U/mL [71]. The above studies prove that it is feasible to use CSL as the medium component of enzyme production to reduce the production cost of cellulase.

### 5.2. Lipase Production by CSL

Lipase is a triacylglyceryl ester hydrolase that has the powerful ability to catalyze hydrolysis and synthesis reactions in nature [72]. It is one of the important varieties of industrial enzyme preparations; it can catalyze reactions such as lipolysis, transesterification, and synthesis. Lipase has great potential applications in the production of biofuels, organic synthetic compounds, detergents, perfumes, food, and feed [73,74]. The synthesis of lipase will be impacted by adding a nitrogen source supply to the medium. Geotrichum candidumrrly-552 was used as the main strain and corn steep liquor was used as the main nitrogen source for fermentation and extracellular lipase production [75]. Under optimal conditions, the maximum production rate of lipase in corn syrup-containing fermentation medium is 0.438 U/mL per hour, which is similar to other common nitrogen sources but less expensive. With corn steep liquor at 2.0%, sesame oil at 2.0%, ammonium dihydrogen phosphate at 0.05%, and disodium hydrogen phosphate at 0.75%, the lipase activity increased 2.16 times to 26.7 U/mL [76]. The medium for lipase production was optimized to use corn steep liquor as the main nitrogen source, and under optimal conditions, the maximum lipase yield in the medium containing corn steep liquor was 0.438 U/mL/h, similar to other common nitrogen sources, but at a lower cost [77].

### 5.3. Protease Production by CSL

Proteases are the most important commercial enzymes for the hydrolysis of peptides and proteins [78,79]. Proteases are ubiquitous in plants, animals, and microorganisms; protease is used most in textile, detergent, leather, feed, and other industries [80]. Protease is in great demand, and the key to protease production is to consider cost and yield. Therefore, it is important to find a more economical and environmentally friendly way to produce protease. To reduce the cost of *Streptomyces* fermentation medium for protease production, feather powder and corn steep liquor were used as the carbon source and nitrogen source, the solid and liquid fermentation methods were used, respectively. The protease yields of these two methods were 21.5 U/g and 13.4 U/g, which increased by 39% and 86% compared to traditional yeast fermentation, respectively [81]. When bagasse (SCB), wheat bran (WB), and corn steep liquor (CSL) were used as a raw materials for a cell–matrix recovery system, the highest protease level (665.5 U/g) was obtained when the CSL proportion was 20% in the same mixture, this process is suitable for commercial production [82].

### 5.4. Other Enzymes Production by CSL

To study the production of endogenous xylanase by *Streptomycetes AMT-3* in Malaysia using by-products from the fermented food industry, the highest xylanase activity was 45.8 U mL^−1^ when wheat bran and CSL were added to the medium. The results show that endoxylanase can also be produced by food industry by-products [83]. Pectin lyase produced by *Aspergillus brasiliensis* in synthetic medium and fermentation of agro-industrial residues was investigated. The highest pectin lyase activity was 300 U/mL in an agricultural medium of 46 g/L orange peel, 160 g/L corn steep liquor, and 150 g/L boiled rice water [84]. The different types of enzymes produced by corn steep liquor fermentation are shown in Table 4.

## 6. Bioconversion of Corn Steep Liquor to Produce Natural Pigment

Pigments have become an important part of our daily life and have a wide range of applications in many fields, such as agriculture, textile, cosmetics, medicine, food, and so on [85]. Numerous natural colors are also recognized as functional substances with possible health advantages. These pigments are frequently used in food, makeup, medication, and other products [86]. At present, pigments are mainly obtained through chemical synthesis and extraction from animal and plant tissues and microorganisms (culture). Compared to plant and animal sources, the production of microbial pigments by fermentation is more economical and can produce biodegradable compounds that can be used in a wide range of industrial applications [87]. In the process of some microbial fermentation to produce natural pigment, strains with high yield can be selected by mutagenesis selection. In terms of medium, changing the composition of the medium and replacing the original carbon and nitrogen sources with low-cost carbon and nitrogen sources for fermentation culture can effectively improve the yield of products and reduce the cost of fermentation [88]. CSL is a concentrated fermented corn extract that is a by-product of the corn milling process and has been used as an ingredient in microbial cultures for pigment production because it contains sufficient organic nitrogen sources.

### 6.1. Lycopene Production by CSL

Lycopene, an unsaturated olefin compound, was first discovered in tomatoes in 1876. Schunck subsequently gave it a name [89]. Red fruits and vegetables contain the lipophilic, unsaturated pigment known as lycopene [90]. Lycopene is valuable because of its vibrant color and lack of toxicity [91]. It has significant uses in the creation of cosmetics [92]. It can also be used as pigment in food processing and is also commonly used as a raw material of antioxidant health foods. The advantages of microbial production of lycopene are higher yields, and the use of inexpensive substrates, such as agro-industrial wastes and residues, to make it more economical [93]. In mass production, the highest concentrations of total carotenoids (1248.5 μg/L) and biomass (7.9 g/L) were obtained within 168 h in medium containing 70 g/L sugarcane molasses and 3.4 g/L corn soaking solution at 25 °C and 180 rpm. To check if this was indeed effective for lycopene production, glycerol was used as a carbon source and supplemented with CSL. Compared with glucose fermentation, the titer and content of lycopene obtained from glycerol and CSL medium were significantly increased by 26.0% (470.6 ± 85.8 mg/L) and 28.0% (138.2 ± 13.9 mg/g DCW), respectively. In addition, output and productivity also increased by 19.4 percent and 25.6 percent, respectively [94]. In conclusion, as an industrial waste, CSL added to the medium can improve the yield of lycopene.

### 6.2. Astaxanthin Production by CSL

Astaxanthin is widely distributed in nature [95]. Many studies have shown that astaxanthin has the potential to fight various diseases, such as cardiovascular disease, cancer [96], and diabetes [97]. Astaxanthin is a kind of carotenoid closely related to astaxanthin, which widely exists in shrimp, crabs, fish, birds, some algae, fungi, and other organisms [98]. At present, the microbial synthesis of astaxanthin is one of the most popular research fields, it has advantages over factory production, and fast-growing microorganisms are easy to grow on inexpensive medium [99]. The macrocomponents, namely the whole mash, corn steep liquor, and glycerol, were fitted by a quadratic polynomial. Under optimized conditions, astaxanthin and β-carotene yields of mixed culture and monocultural culture were 5 and 278, 97, and 275 μg/g, respectively, while β-carotene yields of rose-yeast were 278 μg/g. All of the above experiments demonstrate that the addition of corn steep liquor to the medium as an alternative nitrogen source can increase astaxanthin production [100].

### 6.3. Carotenoid Production by CSL

Carotenoids are the most widely distributed pigments in nature and are found in fungi, algae, plants, and animals [101]. Most animals (including humans) do not synthesize carotenoids from scratch but get them from their diet. Carotenoids are thought to have health benefits [102]. Fungi have great potential to synthesize carotenoids. The most effective source of carotenoids is fungi [103]. In order to increase carotenoid production, many people have tried a variety of methods to produce carotenoids. In order to produce carotenoids from the wild yeasts Rhodotorula mucilaginosa, Sporidiobolus pararoseus, and Pichia fermentans, sugar cane molasses and CSL were used as fermentation mediums, ultimately yielding 830 μg/L of carotenoids [104]. The experimental results show that CSL can be used to obtain carotenoids instead of traditional media. The cell biomass, β-carotene content, and β-carotene produced by adding CSL solution reached 56.32 g/L, 18.92 mg/L, and 60.43%, respectively. β-carotene content increased by 271% compared to the level before addition. This shows that the addition of corn steep liquor to the medium to increase carotenoid production is feasible under laboratory conditions. Different types of natural pigments produced by CSL bioconversion are listed in Table 5.

## 7. Bioconversion of Corn Steep Liquor to Produce Other Novel Functional Components

Protein is an indispensable and vital nutrient for both humans and animals. However, protein resources are still relatively scarce, especially those with special functions, which remain a hot-topic issue in research. Transforming waste proteins into beneficial proteins required by people represents a future development trend. Antifreeze proteins (AFPs) are a class of structurally diverse macromolecules produced by certain vertebrates, plants, fungi, and bacteria. They play a crucial role in the survival of life at sub-zero temperatures and have a wide range of potential practical applications. Antifreeze proteins can lower the freezing point of water in a non-colligative way, while having little impact on the melting point. For example, insect antifreeze proteins often exhibit higher thermal hysteresis than those from fish [105]. In the food industry, antifreeze proteins are used as stabilizers in ice cream, frozen desserts, and other frozen food products. They help to prevent the formation of large ice crystals, maintain the texture and quality of the food, and extend its shelf life. The corn processing by-product of corn steep liquor (CSL) could be used as a growth medium for recombinant Lactococcus lactis, modified to produce antifreeze proteins (AFPs). The corn coproduct media consisting of 50% (*v*/*v*) light steep supplemented in water resulted the best growth and was considered as the best-optimized media to produce antifreeze proteins. The supplemented corn coproduct-based media enhanced the growth of both wild-type and recombinant *L. lactis* strains. CSL can replace or supplement more expensive media, potentially reducing costs. The fermentation supernatants exhibited longer times to supercool and freeze compared to control supernatants, indicating potential use as antifreeze compounds in frozen food and non-food applications [106].

γ-aminobutyric acid (Gamma-aminobutyric acid, GABA) is an important inhibitory neurotransmitter of the central nervous system, which has good water solubility and thermal stability. It has now been confirmed that GABA, as a small-molecular-weight non-protein amino acid, is safe for consumption and can be used in the production of beverages and other foods. Studies have shown that taking a certain amount of GABA has physiological effects such as improving sleep quality and lowering blood pressure in the body. γ-aminobutyric acid was previously produced by the plant enrichment method. Current fermentative production of GABA mainly relies on starch and sugar feedstocks [107]. Xu et al. validated the feasibility of an available non-food lignocellulose feedstock, corncob residue mixed with 30 g/L of CSL (dry base) as nitrogen source, for GABA fermentation. The fermentation parameters in shake flasks and bioreactors were optimized, including the sugar concentration, nitrogen levels, cofactor levels, aeration rate, and fermentation mode. The GABA production was as high as 93.15 g/L from lignocellulosic feedstock and corn steep liquor by-products [108].

## 8. Conclusions

CSL is rich in various nutrients and can be used as a microbial nutritional component in the production of functional components. CSL can be converted into functional components through biotransformation methods. Currently, the more advanced and mature technology is the biological fermentation technology for the production of organic acids, polysaccharides, lipids, enzymes, natural pigments, antifreeze proteins, and γ-aminobutyric acid functional food ingredients. The future development direction of functional food components is to continuously reduce costs, improve the utilization rate of raw materials, and develop products with higher added value. Therefore, the development of functional products through biological fermentation technology and biological fermentation methods is a prominent future research direction. During this process, the metabolic pathways of functional components could be analyzed in future through transcriptome and metabolomics, and the optimization and improvement of the fermentation process play an important role in shortening the production cycle of functional components, increasing the yield and biological conversion rate. In conclusion, the application of corn steep liquor in biotransformation for functional components production has broad prospects for development.

## Figures and Tables

**Table 1:** The main chemical constituents of CSL.

Composition	As a Percentage of CSL	As a Percentage of the Dry Base
Protein	3.19	51.04
Total sugar	0.82	13.12
Starch	—	—
Total acid	1.07	17.12
Ash content	1.17	18.72
Solid	6.25	100

**Table 2:** Types and yields of protein by-products in wet starch processing of corn.

By-Product Type	Corn Gluten Powder	Corn Germ Meal	Corn Steep Liquor
Yield T/(100 T of corn)	6	3.5	8–9.5
Protein content % (*w*/*w*)	60–70	25	45
Current utilization	fodder	fodder	fodder, medium

**Table 3:** This corn steep liquor fermentation produces different types of organic acids.

Name	Organic Acids Type	Amount of CSL Addition	Addition of Other Substances	Result	Reference
Organic acid	Malic acid	5–15 g/L	300 g/L soybean molasses	The PMA yield of soybean shell hydrolysate supplemented with 5 to 15 g/L CSL was significantly higher than that of control supplemented with 2 g/L NH_4_NO_3_.	[22]
	Citric acid	1.0 g/L	60.0 g/L of glucose, 7.5 g/L Na_2_HPO_4_	The citric acid (CA) produced by the strain in the medium containing 1.0 g/L CSL was 27.5 g/L, which was 1.24 times higher than that in the control medium containing yeast extract.	[27]
	Lactic acid	15 g/L	55 g/L lactose, 5.625 g/L ammonium sulfate.	Adding corn steer liquor to ammonium sulfate can increase the yield of lactic acid.	[37]

**Table 4:** Corn steep liquor fermentation produces different types of enzymes.

Name	Species	Amount of CSL Addition	Addition of Other Substances	Result	Reference
Enzyme	Cellulase	1.2% (*w*/*v*)	Brewer’s spent grain 0.5%	The maximum yield of cellulase reached 720 U/L after 4 days of fermentation in the medium containing 0.5% BSG and 1.2% CSL	[68]
		0.19% (*w*/*v*)	Wheat bran 2.0%	When the wheat bran was added with 2.0% and CSL with 0.19%, the cellulase yield reached 2.0 U/mL on the 5th day of fermentation.	[69]
		5.0 g/L	Bagasse 1.0%	The highest enzyme production rate of 1.01 U/mL was achieved at 1.0% bagasse addition and 1.2% corn steep liquor addition.	[70]
	Lipase	8.0% (*w*/*v*)	Soybean oil 0.6%	Extracellular lipase was produced by fermentation with CSL as the main nitrogen source. Under optimal conditions, the maximum production rate of lipase was 0.438 U/mL/h	[75]
		2.0% (*w*/*v*)	Sesame oil 2.0%, ammonium dihydrogen phosphate 0.05%, and disodium hydrogen phosphate 0.75%	*Aspergillus niger* was fermented by CSL to produce lipase. The output of lipase was 26.7 U/mL and the activity of lipase was increased by 2.16 times after optimized medium conditions.	[76]
		15% (*w*/*v*)	Soybean oil concentration	The maximum yield of lipase was 35.20 ± 0.8 U/mL when CSL was used as the nitrogen source.	[77]
	Protease	0.32% (*w*/*v*)	1% Feather meal	Using FM and CSL as carbon and nitrogen sources, the protease production of solid and liquid fermentation was 21.5 U/g and 13.4 U/g, respectively, which were 39% and 86% higher than that of traditional yeast meal fermentation.	[81]
		20% (*w*/*v*)	Food waste 50%, Sugarcane bagasse 10%, Wheat bran 40%,	The highest protease level (665.5 U/g) was obtained when FW 50%, SCB 10%, WB 40% were added to the salt solution and 20% CSL was added to the same mix.	[82]
	Others	1.2% (*w*/*v*)	Wheat bran 2.5%	The highest xylanase activity was 45.8 U·mL^−1^ when wheat bran and corn steep liquor were added to the medium. The results show that endoxylanase can also be produced by by-products of food industry.	[83]
		160 g/L	46 g/L orange peel	Maximum pectin lyase activity was 300 U/mL in agricultural medium (46 g/Lorange peel, 160 g/L corn steep liquor, and 150 g/L steamed rice water)	[84]

**Table 5:** Different types of natural pigments produced by CSL bioconversion.

Name	Species	Amount of CSL Addition	Addition of Other Substances	Result	Reference
Natural pigment	lycopene	20 g/L	50 g/L glycerol and minerals	Compared with glucose fermentation, the titer and content of lycopene obtained by glycerol supplemented with CSL were significantly increased by 26.0% (470.6 mg/L) and 28% (138.2 mg/g DCW), respectively.	[94]
	Astaxanthin	12 g/Lsolids content	32 g/L glucose	CSL has been found to be a valuable and cost-effective supplement to culture *P. rhodozyma* D3, which could both reduce the production cost of astaxanthin and increased the to 1.41 mg/g.	[100]
	Carotenoids	3.4 g/L	70 g/L sugar cane molasses	Using agricultural medium (6.5 g/L corn soaking solution and 30 g/L sugarcane molasses) for feeding batch fermentation, 830.3 μg/L ± 27.0 μg/L carotenoid was obtained, CSL can replace traditional medium to obtain carotenoids.	[104]
		20 g/L	40 g/L Glucose, 1 g/L K_2_HPO_4_ and KH_2_PO_4_, 0.05 g/L FeSO_4_.	Cell biomass of CSL solution at 0–5% dissolved oxygen saturation, β-carotene content increased by 271% compared to the level before addition.The biomass was 56.32 ± 0.5 g/L, and the β-carotene content was 18.92 ± 0.3 mg/L.	[84]

## Data Availability

The original contributions presented in the study are included in the article, further inquiries can be directed to the corresponding author.
